# Championing Equity, Empowerment, and Transformational Leadership in (Mental Health) Research Partnerships: Aligning Collaborative Work With the Global Development Agenda

**DOI:** 10.3389/fpsyt.2019.00099

**Published:** 2019-03-13

**Authors:** Manasi Kumar

**Affiliations:** ^1^Department of Psychiatry, University of Nairobi, Nairobi, Kenya; ^2^Department of Psychology, University College London, London, United Kingdom

**Keywords:** global north, global South, global mental health, development, empowerment, equity

## Abstract

Through a narrative synthesis of existing literature on research partnerships, the paper underscores four core values championed in public policy and practice: equity, empowerment, transformational leadership, and treating mental health research as a cooperative inquiry. Building on these values, the author maps the challenges before mental health researchers in forging resilient, egalitarian, and committed Global North-South partnerships within the context of current global development agenda. Reports appraising the UN Millennium Development Goals lament how the goal of developing global partnerships to combat health, gender, and economic inequities has remained under-realized. Emphasis has been placed on the great need to augment Sustainable Development Goals (SDG) in ways where partnership processes would drive development and human rights agenda for the most afflicted, under-resourced, and marginalized in the world. Global North-South partnerships result in fewer lasting benefits to Global South-a regressive trend that is critically analyzed. The need for Global North to adopt ethical and responsible stances while creating/curating new knowledge is discussed. Being responsible is not only imperative for Global North researchers; it is imperative for both North and South researchers to adopt a dialogical approach in clarifying and sharing roles, responsibilities, access, and leadership in developing scholarship and praxis in mental health. The importance of de-centering hierarchies, valuing reciprocity in one another, improving communication, demonstrating empathy, and sharing resources and benefits are found to be key components in the narrative synthesis towards achieving greater empowerment and equity. The paper reflects on the potential problems in engagement and development of de-centered and transformational leadership in partnerships and implications for research ethics in the context of lower-and-middle-income countries. Lastly, the author in a bid to encourage global partnerships suggests that engaging in transparent and bi-directional conversations regarding these issues and realigning research priorities along the four core values will contribute to greater success in research collaborations (across cultural contexts) and more so in the global mental health field.

## Global Research Partnerships: A Panacea of Variable Practices and Expectations

Partnerships are the driving force to attain health equity in both high- and low- income countries where access to quality and timely care continues to be a development issue (1). Highly vulnerable populations, whether these be marginalized communities; children and youth; aging populations; communities and individuals stigmatized and socially excluded for their sexual orientation, beliefs, and practices; those living in poverty and socially disenfranchised have been groups carefully studied under the remit of global research partnerships (2, 3). The premise of collaborative work is that these communities and special populations are tainted across the board and only in working together can remedial strategies be discerned.

Global health partnerships have existed for as long as medicine, technology, and research has allowed cross-cultural contact and networks. Subsumed within these partnerships are efforts at addressing global health inequities. The concept of the right to health underpins all such international advocacy such as the International Covenant on Economic, Social, and Cultural Rights (4). Several conceptual and theoretical frameworks have tried to address inequities and mobilized efforts and resources to address these issues. Social determinants of heath framework have become an insightful and offer a vast repertoire of compelling evidence on conditions that impact human well-being and health (5, 6). The framework epitomizes the value of transdisciplinary engagement with different knowledge spheres by using different levels of analysis for different levels of stakeholders (7). Global health modeling initiatives like Global Burden of Diseases use a transdisciplinary partnership approach where disease and corresponding relative harm estimates give a fuller picture of health of a region or population. Additionally, they estimate associated social determinants impacting these conditions; so that the global community can direct its efforts in ranking diseases and populations within and across the globe. So that the research and policy priorities can be developed in a more focused manner (8).

UN Millennium Development Goals were heavily critiqued for lacking clear indicators on research and on international and policy partnerships on non-communicable diseases (NCDs) (9). Sustainable Development Goals (SDGs) have had to focus on partnerships as a vehicle for enforcing development agenda and policy refinement and have incorporated NCDs in a more refined manner (10). The goals 10 and 17 most lucidly tackle these problems. *Goal 10 addresses the ways to strengthen the means of implementation and revitalize the global partnership for sustainable development and goal 17 focuses on promotion of peaceful and inclusive societies for sustainable development and provide access to justice to all and build effective, accountable and inclusive institutions at all levels* (11, 12). The SDGs underscore the interdependence between Global North and Global South and push the momentum toward interrogating structural and health disparities. Despite this common ground, researchers have noted that the fundamental flaw in the SDGs may be their implicit assumption that the same economic system, with its still-present neoliberal governing rules, that led to the current era of severe inequality and environmental peril can somehow be harnessed to engineer the reverse, however vehemently international health-rights promoters would advocate such a model of change (13–15). “What should a health promotion ethos and practice look like in an era of anthropogenic depredation, economic stagnation, and a “liquid modernity” that challenges the possibility of collective politics, where one can all too easily devolve all ethical or social responsibility to the individual?” (13, p. 675). Other commentators have said that SDGs are a global statement about *the world we would like to live in* rather than the world and the realpolitik we inhabit currently. SDGs have been imbued in a more hopeful, positive, and even more forceful framework than any multilateral diplomatic framework around development (12) and yet the idea that the current systems could be utilized to process this change is a bitter pill to digest.

Whatever little global health partnership literature there exists, the corpus strongly advocates development of practice-research community (16). Researchers, health-system practitioners, and other governmental, non-governmental, and community stakeholders need to come together in *an almost deliberate act of cooperation and collectivity to devise research and policy strategies*. The need to be an agent of change in whichever identity we around is critical to the process of collaboration. At the heart of this paper are concerns around how do we create equitable and collaborative partnerships to bolster sustainable development, how are these collaborative processes conceptualized, and which common codes of conduct can be dialogically created when it comes to global mental health research. Which groups benefit to what extent matters ethically (17), especially how do partners envision and carve out their roles collaboratively and the ways in which these roles pan out over a period of time are important. Within global health, global mental health is clearly a field where cost and time-effectiveness of interventions and approaches matter, we also know that cost-effective or time-effective do not necessary imply equitable allocation (be it of human or material resources). This also brings in a related quandary: without developing complementary practical guidance involving different stakeholders, the process of collaboration, knowledge generation, and health equity may remain inchoate.

## Global Mental Health: Etching out the Argument for Equity, Empowerment, Capacity Building, and Systems Strengthening

Various global conventions (Alma Ata of 1978, MDGs to current SDGs, WHO Right to Health) ask international collaborative research in mental health to consider local needs and developmental trajectory of the communities in resource-constrained contexts. Both the WHO mental health treatment gap action plan (18) and Global Mental Health Action Plan 2013–2020 (19) were a response to implementation and service gaps. Both these plans have endorsed utilizing *a developmental approach*—reviewing the systems and dynamic interactions between various agents as they are evolving and *a collaborative evaluation approach* which seeks to include various stakeholders in reviewing and critiquing the process and outcomes of mental health system strengthening (20). A number of studies have pointed to the epistemic (21) and practical ways (22) in which mental health capacity building can take place. The capacity building efforts can be marred by the old ghosts (15) which need to be tackled effectively with efforts from all partners.

## Equity and Empowerment: Laying out the Problematics

1. *Issues of participation and ownership of research being conducted in the Global South*. The research belongs first to the people on whom the study was carried out. The ownership of the research must belong to the studied participants, researchers, and communities (22). How this process shapes is a learning curve in life of a mental health or implementation researcher but there should always be clarity around the question of agentive identity and entitlement here. Taking the process work and study outcomes back to the participants not once but in a systematic, protracted manner, enables community, social, and systems change to get into motion. Several capacity building studies have pointed to the absence of rigorous research and evaluation methods, limiting an in-depth understanding of mental health systems strengthening and effects of associated contextual factors (23).
2. *Issues of power, transparency, process accountability, structural disparities etc*. In forthcoming sections, dynamics of oppression and subjugation have been laid out from a critical theory point of view (see Spivak, Said, Foucault for more nuanced analysis of these thematics). The capabilities framework (24, 25) has also been utilized to underscore the absence of freedoms and choices before the Global South^1^ researchers and the genuine need to empower and strengthen human capabilities in mental health research. Power is the piece that connects oppression to disparities and inequity. Transparency about intentions, process, and benefit-sharing are values in research that need to be collaboratively enforced. Democratic communication and non-hierarchical relationships between experienced, senior colleagues and students, junior mentees, or early-career researchers both from Global North and South create opportunities for mutual learning and new pathways for research growth. There may be differences in what one wants from the partnership or even minor to significant differences in what matters to one group over the other, these differences should branch out from a position of reciprocity. Reciprocity is the minimum goodwill condition to foster prosocial and equitable practices.
3. *Factoring equity not only between researchers from Global North and South but also those within Global South or North; also factoring equity in recognizing contextual factors that trigger oppression*. Given the inequalities and structural disadvantages that global South encounters, the rhetoric of equality in mental health research partnerships takes away the opportunity to critically address these problems (28). While much of the paper is a commentary on the multilayered nature of Global North and South partnerships, the issues around equity also touch on disparities in human rights, entitlements, and health within minoritized groups, sub-populations within Global North and South. Poverty or experiences of marginalization based on ethnicity, race, or sexual orientation are not unknown to Global North or to Global South. Addressing disparities within one's own context is critical. There are unique contextual factors associated with disparities in the two hemispheres that need to be carefully studied. In this regard, researchers from both the contexts have to be “consumers and producers of the research” (15). One connected idea is that few initiatives within global mental health have promoted Global South-South cooperation and partnership so that lessons can be shared and common social determinants including, common risk factors associated with the burden of mental disorders could be addressed. Only South-South dialogue can determine which elements of global development agenda are suitable (29) and in which directions would they need to etch out a completely new path with new set of indicators and goals.
4. *Understanding issues from an experiential, phenomenological and critical standpoint, especially in non-communicable diseases and mental health*. Global mental health literature at times becomes replete with epidemiological, methodological, and complex jargons. Whilst methodological and theoretical refinement in layering the issues is important, lack of emphases on understanding political, human, and material resources and access to resources related to dialogue around health and mental health specifically, is really missing. There is a need to develop phenomenological approaches derived from local contexts (be it in Global North or South) to lend voice to the most marginalized, to give shape and contour to distress emanating from social isolation and other adverse social determinants and to explain psychological maladies in more experiential ways that are not boxed under universalist diagnostic guidelines. The tendency to reduce the “global” to a few categories has to be replaced with greater dimensional thinking (15, 30).
5. *Global South's role of data mongering for extending careers of people in Global North*. The Global North researchers are often in possession of more economic, symbolic, and social capital and the Global South researchers possess more cultural capital which needs to be utilized in innovative and porous ways to change the more entrenched disparities. In utilizing Bourdieu's work, Walsh et al. (31) argue that research collaborations should be seen as projects in developing a sound ethical code of conduct. These partnerships should aim to balance knowledge, interest, and power in the short term, with the aim of maximizing benefits for LMICs in the longer term. Considerable literature now reflects on the problematics associated with publications and senior authorship credits where the contributions of Southern researchers are undermined. It is well-known that deficiency in authorship policies, perceptions of bias, and cultural differences contribute toward questionable or unethical authorship practices (30, 31). The difference between procedural ethics and ethics in practice is noteworthy in shaping the researcher's identity. Simon and Mosaval (30) argue for development of an “ethical mindfulness” where resources, benefits, and access to opportunities are equitably distributed and shared. The asymmetry between the procedural ethics and ethics in practice creates dissonance and leads to a greater subjugation of the subaltern in some ways. Following observations can be made about this asymmetry.

## why Research Partnerships Need to be Viewed as a Co-Operative Inquiry?

1. *Partnerships sometimes are built at the cost of rights of individual research participants and collaborators:* the process of retrieving individual voices and ideas in a reciprocal manner should be an ethic in mental health research. Psychology and behavioral sciences are known for their “zeitgeist” fervor where theoretical innovation was always regarded paramount. Retrieving individual voices and concerns in a bid to promote individual team members, knowing the strengths and contributions of each team member would only build the collaboration further. In order to advance a high quality mental health research, the chiseling of personal integrity, self-reflection, and shared ownership of the process is critical (32).
2. *Lack of local, national regulatory bodies, and competent research governance:* in the absence of regulatory bodies and effective mental health system governance, the effort should be toward systems capacity building in any mental health implementation work. For the LMIC context, where mental health may not become immediately high priority for national governments, strengthening mental health systems from lessons learnt through national level policies is critical. A recent mental health policy review demonstrated that capacity building can be vexed if carried out from the apertures of high-income partner/global North experiences entirely (29). The unique challenges, opportunities for more radical reform and the dynamism needed to optimize limited resources for the cause of mental health demands a more careful scrutiny of national and regional policy planning. Over and beyond the critical grasp of national policy guidelines, the coordination, strengthening, and enforcement of local mental health laws would be imperative (15). The mental health regulation and governance can only be effective if it is not an implant from outside but germane to the existing health services implementation challenges. WHO Assessment Instruments for Mental Health Systems (AIMS) and mental health ATLAS are one such resource to empower national level policy and systems development that needs to be embraced by countries where such systems are underdeveloped or non-existent (33, 34). The relative lack of demand and capacity for priority setting in global mental health in LMIC is a concern (35). The priority setting in global context has been top-down and with the WHO prioritization of a bottom-up approach this can be rectified. Additionally, the focus of policy, system and services has to become more *horizontal* than increasingly more verticalized (17).
3. *The needs, agendas and foci vary between the North and the South partners. Relationality and reciprocity are the key to develop a minimum common program:* different partners enter research with different values, aspirations, and skill-sets. The expectations vary enormously. However, the connection that binds the collaborative work has to stem from reciprocity and relationality. The notion of “ethical mindfulness” creates a meditative and reflexive stance around issues and processes that need to be co-opted and developed. Notions of researcher “reflexivity” and “transference and counter-transference” from the fields of psychological research and psychotherapy map processes that cannot be immediately captured in words or speech but need a little pause and reflection. Reflexivity allows an appreciation of the researcher's stance, background, or baggage and transference and countertransference taps into the unconscious mechanisms that build a relationship between two researchers (traditionally between client and therapist or participant and researcher). Both these mechanisms build relationality and reciprocity into the research equation. Reciprocity and relationality may include asking the other what they think could be the problem and how a solution could be arrived at (31, 32).

## Problematics of Ownership, Entitlement, and Liberation: the Challenge of Global North

The researchers in the North have a pressure to produce scholarship that is constantly game-changing, innovative, and original. In this process, the researchers face pressure to generate resources for their own survival as well as cover institutional overheads to support the University administrative machinery which can be a trying enterprise. Partnerships therefore are strategic, tactical, and are aimed at benefiting the end point which is being successful in grantsmanship, scholarship, and generating high impact from the partnership. The imagination of “Global South” as a lucrative site for intervention does not always imply a careful rethink of power, agency, or even true meaning of collaborative work. Re-engineering a neocolonial strategy by bringing skills, resources, and solutions, the Northern researchers descend on the South. Spivak (36) refers to field work by North researchers in the Global South as being part of “information retrieval” leading at times to recreating (neo)cultural imperialism in some ways. The obsession with productivity means that the eye is constantly on the final product or outcome and less frequently on the process and mechanisms to arrive at the outcome. Imbued in what appears to be a self-promotional agenda that focuses on bolstering Northern institutional resources, these researchers become engaged in partnerships that are largely self-serving. Equity and empowerment when it comes to tangible outcomes are neither aspirations nor mandatory in arriving at the endpoint (10, 31).

Northern researchers juggle with other intersectional identities in order to blend in and be accepted in their milieu. These researchers shed aside their ethnic or cultural differences to the comply with a universal code of research engagement and partnership. This stance involves *de-individuation* (37) and *obedience to authority* (38) both of which might keep benefit-sharing, transparency, and social justice at bay. Given that the funding and resources are largely managed by Western funding agencies, the protectionist policies safeguard the interest of the North. Additionally, “the hand that gives” also reduces collaborative agency, a fact that cannot be ignored. The research done in most partnerships extend research careers and political agendas of researchers in the global North. The real capacities of the Southerners are not developed fully consequently neither is the bilateral or multilateral research experience fully optimized nor any effective system or skill-sets strengthening takes place.

This is not a banter against more able and resourceful factions versus the less privileged ones. The intersectional nature of researcher identity has been studied in a variety of disciplines. In her essay on *Can the subaltern speak?* Spivak (36) reminds us of the proclivity of dominant discourses and institutions to marginalize and disempower the Third World “subaltern”^2^. Reminding us of Foucault and Said (41), of the objectification of the non-Western world, Spivak reminds us that the interactions with the subaltern are rarely at the same level playing field (42). Postcolonial studies in their interrogation of feminism pointed out to the two signatures of the feminism: one emanating from Global South and the feminism from “third world feminists” residing in the first or second world (43). The “third world feminists” often spoke on behalf of the Southern feminists and their own feeling of oppression and marginalization in the North was a subtext (unconsciously leaked into the narrative) that was never fully decoded. Spivak reminds us about the disputable claims to knowledge, representation, and authenticity in writings of third-world researchers based in the first world, who do not interrogate problematic practices in their own work or in their engagement with the subaltern or the Global South. There is an uncomfortable positioning of the research “subject” in the work of “third world researchers” in first or second world and they are therefore both victims and complicit in the politics of oppression. The discourse on equity enters right here. Academic cultural imperialism is then a process of offering a “bait” to the Southern researcher to turn “information,” “selected facts” into a well-oiled machinery of knowledge production. To learn to empathize with the position of the “informer”-that is the nativist or the subaltern, should be a role-training the Northern researchers must have. Knowledge is imbued with power and the temptation before the Global South researchers for their assistance in field work to resolve their own problems and become more empowered is always present. Another uncomfortable truth exists around the complicity with which structural problematics are attended to: hierarchies, gender, race inequalities are not necessarily challenged by the Global North but maintained in the way power and its vagaries are preserved. The true issues of the South are represented, led, and analyzed without any compunction by Global North researchers. The relationship with processes of authenticity, representation, and accountability—are quite vexed in this regard.

Just as the identity of the second or third world researcher (immigrant settled in the first world) in a first world global mental health context is vexed (there are also accompanying excitements and opportunities that cannot be understated), the first world researcher (of whatever racial, gender or other ethnic profile) becomes very prescriptive in the way he or she would visualize the growth trajectory of a Southern researcher but keeps a lot of leeway in planning their own journeys. The Southern researcher would seem to be very ambitious if working in multiple contexts or framing work in global context, however these domains lend themselves naturally to a researcher representing the North.

## Psychology of the Oppressed and Oppression: The Challenge of the Global South

The South is the site for perpetual “innovation” and “intervention” and in theory both North and South researchers earn their rank and file building resourceful and robust collaborations geared toward solving massive global health problems. Many-a-times these partnerships are shaped by “Othering” the human condition, structural inequalities, and at times also the research participants. The process of engagement with global health research disconnects researchers from a critical discourse on structural inequalities and the material causes of health disparities. The researchers stop interrogating the problematic practices. Fanon in his analysis of the dialectical oppressed-oppressor identities makes an important point about the psychology of the subaltern. Oppression is a process of subordinate and dominant identities. The psychology of oppression is a “way of understanding psychological debilities as products of social oppression” (44). In the throes of various kinds of structural deficits, the Global South researchers do not necessarily come together to build and develop their own capabilities. Borrowing from Sen (25, 45, 46) capabilities are people's valuable functionings and functionings are the state of *being and doing*. According to Sen (25) and Nussbaum (47), a person's capability represents the effective freedom of an individual to choose between different functioning combinations—between different kinds of life—that she or he has reason to value. Both functioning and the freedom to choose are negatively affected in the Global South and individual agentive functioning becomes thinner in such collaborative work. If Sen's capabilities would provide freedom from domination, then mental health advocacy within such a framework, would organically promote development as social justice.

Global South is also known for its rigidities and adherence to varied hierarchies: seniority prevents researchers from engagement with younger early-career researchers, tribalism prevents more able leadership to develop, hoarding resources, and benefit accumulation prevents benefits to be equitably shared. The socio-political set up neglects the considerable talent and creative thinking and does not engage with innovative ideas (48). The investment in youth has been so poor in global public health and this also reflects in the state of child and adolescent mental health in the fields of psychiatry and psychology in the Sub-Saharan Africa.

The psychology of the oppressed also includes the inability to share, show concern, and trust one another (those from one's own milieu). Internal factions, tribalism, gender divides, and racial politics does not unite Global South researchers but keeps them hustling with each other. Power hoarding creates subordinate structures and stagnant narratives. Where the Global South researchers dominate global mental or public health discourse, the fight is for resources and fame that comes by facilitating the “field work” of the Global North researchers or becoming complicit in other ways such as using subordinate colleagues or communities with little awareness or empowerment to keep the real concerns on the discussion table. If only the South believed in “reverse innovation” many small experiments in its own tropics and Saharas would have had greater impact and resonance. The self-doubt, insecurity of attachment to one's own and cultural cause at times, creates impasses in this journey toward more forceful egalitarian worldview that the Global South can lend voice to. The preponderance of the experience of “relative deprivation” when mapping the capabilities of Global North over Global South (5, 6) would need to become an awareness in global mental health researchers not only for understanding heath disparities but to also target capacity building in the right direction.

The section above alluded to the representation of global mental health concerns from the South emanating from voices of the third world researchers based in the Global North and those located in Global South. There is the real South downwards of the Northern hemisphere and then there is an imaginary play and representation of South in Global North by those who seem to be affected by similar politics of seclusion. Both these voices are legitimate but may be driven by different agendas, needs, and politics of marginalization. We have to tread carefully as we may make voices of the most marginalized and mentally afflicted populations of Global South if we use “South” as a trope rather than as a way of opening the discussion to everyday oppression and marginalization which exists in both locales.

The Oxford dictionary defines “*mafias*” as “an organized international body of criminals, and having a complex and ruthless behavioral code.” In an otherwise non-literal definition the Cambridge dictionary says *it also alludes to a close group of people who are involved in similar activities and who help and protect each other, sometimes to the disadvantage of others*. The global health mafias are these power hubs and these institutionalized systems where subordinate and dominance cycles continue to thrive and perpetuate the message of oppression. Both the hemispheres are susceptible to these and are affected by this internally as well as through contact with each other.

## Movement Toward Equity and Empowerment: A Mental Health Science That Cares for Social Justice

UNESCO Declaration on Bioethics and Human Rights (2005) is the most comprehensive guideline on benefit sharing in research. Benefit sharing includes access to the intervention being doled out, provision of health care for participants, capacity building for individuals and institutions, support to health care systems, and access to medical and public health interventions proven effective (49). There is also a wide berth for who is regarded as beneficiaries. These can be research participants, their communities or those affected, researchers from these contexts and there continues a debate internationally as to which beneficiaries need to be prioritized. Whatever the differences may be, the impetus needs to be on the social value of research and translating research into improved health especially for low-resource context communities. In framing the work closer to needs and ideas of various stakeholders on ground, the social value and justice could be achieved (50, 51).

Equity is denoted by authentic partnering, inclusion, shared benefits, humility, responsiveness to the causes of inequalities, and commitment to the future. In keeping equity at the heart of mental health research enterprise, capacity building of individuals and institutions becomes an ethic in itself (50). We should differentiate between pseudo-empowerment and real equitable partnership. This is the difference between “feeling” empowered and “appearing” to be empowered—something which is gleaned from outside so to speak (52). There is a shared learning process in true empowerment where one may begin with individual learning but takes it to joint institutional learning. Various blended learning designs have developed a methodology for this process (53).

Social justice perspective seeks to interrogate monolithic notions of knowledge and commitment through adoption of a human development approach (Sen and Nussbaum's work in particular). The social justice perspective utilizes the health capabilities framework extended by the work of Amartya Sen. In this paradigm, the priority is given to focusing on the health needs of those farthest from the optimal level of health (35). Two models guide this framework: *shared health governance model* (talks about collective ethical commitment to equity in health) and *functional requirements principal* [allocation of specific duties to specific national and subnational actors with the assumption that duties be equitably distributed so that the respective institutions or individuals could provide those functions (54, 55)]. By enabling our research participants and co-researchers partake a “voluntary commitment” in mental health research the ethical norm of health equity is internalized at institutional, group, and individual levels. Shared governance becomes like an entitlement just as health equity is an entitlement for individuals and communities at all levels in this framework. The social justice model incorporates three values of—priority setting, capacity building and post-study benefits sharing that must underpin all research enterprise to attain health equity. The focus on NCDs in the global burden of disease is one step toward addressing health in social justice context. Within the NCDs, the focus on global or public mental health is a process of accounting for the capabilities-deprivation that adverse social determinants leave vulnerable populations with.

Social justice frameworks demand the researcher to not be of a Skinner's “black box” alone but have appreciation for history, culture, and polity of a particular context from where they come and where they practice or carry out field work or data collection. This is more relevant for those researchers who passionately (often times naively) transcend geographical borders to study mental health in populations unknown to them. The temptation of testing Western models, of using a common theories approach to universalize and streamline complexities may undermine the deeper nuances of praxis associated with integrated culturally rooted mental health in an instrumental manner.

In a nutshell, the capabilities framework in principal is about individual freedoms and should enhance researchers' (and people's, communities') abilities and freedoms to make choices and uphold their ideas. Once such an ethic is imbibed, mental health researchers would in some ways act like human rights advocates and frame mental health as a human right which is essential for equity and democracy.

## Transformational Leadership: What Does That Mean for Mental Health Partnerships

Capacity mapping and resilience are discourses that a transformational leadership considers at the core their enterprise. Leaders who will bring transformation would be agile, having done their homework around mental health systems and have a great understanding of the context and its champions, participants, and researchers as well. A transformational leadership creates a “sufficientarian” threshold (47) of minimum requirements of entitlements and capabilities that could be claimed by the community of researchers and participants engaged in a collaborative study process. The exact location of this threshold is not a monolith but an agreement that is democratically debated and incorporated into research, community, and even deliberated further in the national constitutional, international human rights, and international development processes (47).

A transformational leadership is very receptive about the political issues emanating from the field, keen to map the system needs from a wide geopolitical berth. To this end, such a leader learns from the stakeholder feedback without being flummoxed or being challenged. The capacity to mentalize the political while thinking of individual distress amelioration is what transformation in mental health systems and governance is all about. Such a leadership extends rights, development, and mental health agenda. This is also about a leadership that keeps innovation and also possibilities of “reverse innovation”—an idea that the innovation in Global South would help reduce mental health and social determinants related inequities and disparities in the North, in sight (52). It is not enough to merely visualize “reverse innovation,” efforts have to be made to demonstrate how this reversal took place and what translational lessons were learnt. The institutional level lessons learnt in pursuit of reverse innovation and knowledge-technology transfer will provide great anchorage to the overall success of mental health partnerships that a transformational leadership factor, mindfully. The process of transformation in a leadership context also includes operationalizing engagement [both consenting and community engagement, (56)] so that best practices can develop collaboratively. A good leader, especially so in mental health research, would be someone who is able to steer partnerships and collaborative practice-based research from more short term, interventionist, coordination types to a more long-term, integrated implementation vision (16, 52). This particular type of leadership is not gender-or -race neutral, it seeks diversity, representation, and empowerment of men and women, young and old to consolidate partnerships and collaborative work. The absence of women especially women (and men) of color representation in mental health in diverse geopolitical locales of Global South demands greater participation and gender-mainstreaming not just as a lip-service but as a way of questioning historical past of racial and gender oppression. An engagement with sexual, racial, ethnic minoritized groups, with younger people who are largely undervalued in Global South, a transformational leadership does not eschew diversity or difference. Postcolonial perspectives whether from philosophy of sciences, ethics, literature or poverty studies, could potentially enrich the mental health partnerships and international research discourse. Both difference between text and meaning, practice and theory, lived experience vs. imagined reality matter in a geopolitical context with history of oppression to introduce perspectives from neoliberal, globalized world order without critical or historically annotated goal posts, would make this homogenizing of mental illness idioms hugely problematic. The transformational leadership should be imbued in thinking about how capabilities be fostered given a particular context of oppression, limitation, or resource constrain. The creation of opportunity role structures that are opportunities to participate in valued social roles in global South context specifically could introduce both the researchers and participants with new dimensions associated with freedom and social choice (57).

## Conclusion

This narrative review and synthesis paper offers a scrutiny of critical ethical and research engagement issues that affect global mental health partnerships and international health collaborative work. The solutions are not easy and require a highly reflexive and well-thought out response to researcher's geopolitical, social, economic, and cultural differences. These differences are etched onto the Global North and South divide. A leadership that is transformational and not transactional, a process that is relational, reciprocal yet transparent, fair and equity promoting is the way out if we truly want to strengthen the field of global mental health.

## Author Contributions

The author confirms being the sole contributor of this work and has approved it for publication.

### Conflict of Interest Statement

The author declares that the research was conducted in the absence of any commercial or financial relationships that could be construed as a potential conflict of interest.

## Footnotes

^1^Global North and South may appear like grand narratives for what may be more complex political, geographic, and economic factions. There are several definitions available, here the global South refers to the transnational political subjectivity under contemporary capitalist globalization and draws from the rhetoric of the so-called Third World Project, or the non-aligned and radical internationalist discourses of the Cold War (26). The global North typically comprises of the first world countries, sometimes alludes to the G8 or European and North American contexts, some argue North covers both the first and the second world including the permanent members of the UN security council (27). There are many Souths in the North and many Norths in the South- one may exist in the other.

^2^The term subaltern denotes a person holding a subordinate or inferior position. It was first used by the Marxist theorist Antonio Gramsci to explain the concept of cultural hegemony. However, in the later work of critical theorists and historians it is used as a general attribute of subordination that cuts across class, gender, race etc divides (39). Another proposition has been to read *the subaltern* as a denigration of the concept of agency and denying the role and impact of social structures (40).
